# Are morphological criteria sufficient for the identification of circulating tumor cells in renal cancer?

**DOI:** 10.1186/1479-5876-11-214

**Published:** 2013-09-17

**Authors:** Amin El-Heliebi, Thomas Kroneis, Evelyn Zöhrer, Johannes Haybaeck, Katja Fischereder, Karin Kampel-Kettner, Richard Zigeuner, Hannelore Pock, Regina Riedl, Rudolf Stauber, Jochen Bernd Geigl, Berthold Huppertz, Peter Sedlmayr, Carolin Lackner

**Affiliations:** 1 Institute of Cell Biology, Histology & Embryology, Medical University of Graz, Harrachgasse 21/VII, Graz, 8010, Austria; 2 Institute of Pathology, Medical University of Graz, Auenbruggerplatz 25, Graz, 8036, Austria; 3 Department of Urology, Medical University of Graz, Auenbruggerplatz 5/6, Graz, 8036, Austria; 4 Department of Internal Medicine, Division of Gastroenterology and Hepatology, Medical University of Graz, Auenbruggerplatz 15, Graz, 8036, Austria; 5 Institute for Medical Informatics, Statistics and Documentation, Medical University of Graz, Auenbruggerplatz 2, Graz, 8036, Austria; 6 Institute of Human Genetics, Medical University of Graz, Harrachgasse 21/VIII, Graz, 8010, Austria

**Keywords:** Circulating tumor cells, Circulating tumor microemboli, Renal cancer, ScreenCell®, Array comparative genomic hybridization (array-CGH), Circulating endothelial cells

## Abstract

**Background:**

Single circulating tumor cells (CTCs) or circulating tumor microemboli (CTMs) are potential biomarkers of renal cell cancer (RCC), however studies of CTCs/CTMs in RCC are limited. In this pilot study we aimed to evaluate a novel blood filtration technique suited for cytomorphological classification, immunocytochemical and molecular characterization of filtered, so called circulating non-hematologic cells (CNHCs) - putative CTCs/CTMs - in patients with RCC.

**Methods:**

Blood of 40 patients with renal tumors was subjected to ScreenCell® filtration. CNHCs were classified according to cytomorphological criteria. Immunocytochemical analysis was performed with antibodies against CD45, CD31 and carbonic anhydrase IX (CAIX, a RCC marker). DNA of selected CNHCs and respective primary tumors was analysed by array-CGH.

**Results:**

CNHC-clusters with malignant or uncertain malignant cytomorphological features - putative CTMs - were negative for CD45, positive for CD31, while only 6% were CAIX positive. Array-CGH revealed that 83% of malignant and uncertain malignant cells did represent with a balanced genome whereas 17% presented genomic DNA imbalances which did not match the aberrations of the primary tumors. Putative single CTCs were negative for CD45, 33% were positive for CD31 and 56% were positive for CAIX.

**Conclusions:**

The majority of CNHC-clusters, putative CTMs, retrieved by ScreenCell® filtration may be of endothelial origin. Morphological criteria seem to be insufficient to distinguish malignant from non-malignant cells in renal cancer.

## Background

Hematogenous dissemination of single tumor cells (circulating tumor cells, CTCs) or tumor microemboli (circulating tumor microemboli, CTM) is an important mechanism involved in the formation of metastases, the major cause of cancer related death [1]. Results of numerous studies indicate the prognostic value of CTC detection and enumeration in many human cancers. This has been confirmed in recent meta analyses for breast [2] and colorectal cancer [3]. Besides enumeration, results of the molecular analyses of CTCs may be used to predict treatment response [4]. Investigation of CTCs may hold great promise to inform individualized treatment strategies as well as to increase the knowledge about the metastatic process in itself. Renal cell cancer (RCC) is the most frequent solid lesion of the kidney and accounts for 3% of all cancer cases worldwide. Approximately 20% of primary localized RCC will develop metastatic disease negatively impacting on patient survival [5]. Therapeutic options are limited by resistance of RCC to chemotherapy and radiation, but have recently been improved by the advent of targeted therapies [6,7]. As in other malignancies, CTCs may also be valuable prognostic and predictive biomarkers of renal cancers, however studies of CTCs in RCC are limited (reviewed in [8]).

Several techniques for the detection and enumeration of CTCs have been developed during the last years and the list is continuously growing. Many methods use epithelial antigenic properties of cancer cells to detect and isolate them from blood by immunomagnetic or microfluidic based enrichment methods (reviewed in [9]). However, many of these detection systems are not commercially available and/or economically accessible. Another obstacle is the epithelial antigen-based detection of CTCs. Epithelial to mesenchymal transition (EMT) is believed to represent an integral component of the metastatic process in which cancer cells down regulate the expression of epithelial markers in favour of mesenchymal markers, a process linked to the stemness of cancer cells and increased chemoresistance [10-13]. Hence such CTCs may therefore escape detection. Recently filtration based size exclusion technologies have been developed [14,15] which allow for antigen-independent isolation of CTCs from blood based on their larger size and cytomorphological features in comparison to hematological cells [16]. Some of these methods like the ScreenCell® filtration system are commercially available. CTCs can be isolated from diluted blood in a single step using a translucent polycarbonate membrane. Following filtration they can be further analysed by light microscopy and immunocytochemistry [16-21] but molecular data on putative CTCs/CTMs are limited [21-23].

Using the ScreenCell® filtration system and array-CGH technology we aimed to evaluate if morphological criteria [17] are sufficient to identify CTCs/CTMs in blood of renal cancer patients. Here we report for the first time chromosomal analysis of circulating non-hematological cell clusters cytomorphologically resembling CTMs by array-CGH.

## Methods

### Ethical statement

The study was approved by the ethics committee of the Medical University of Graz (reference EK: 19–239 ex 07/08). Written informed consent was obtained from all patients. The human iliac arterial endothelial primary cells (HIAEC) (generously provided by Dr. I. Lang-Olip, Medical University of Graz, Austria) derived from an organ donor. Ethical approval was granted by the ethics committee of the Medical University of Graz (reference EK: 19–293 ex 07/08). The ethics committee waived the need for written informed consent as the donor fulfilled presumed-consent according to Austrian Hospitals Act.

### Patients and tissues

Thirty consecutive patients diagnosed of renal cell carcinomas (25 clear cell and 5 papillary carcinomas) and for control purposes 10 patients with benign renal tumors (1 renal cyst, 2 angiomyolipoma, 6 oncocytoma and 1 cystic nephroma) diagnosed between 2010 and 2011 were included in the study. All patients underwent surgical resection of the tumor. Clinical and histopathological parameters of the study cohort are compiled in Table 1. Blood samples from 20 healthy volunteers were used as age and sex matched negative controls. Formaldehyde (4% m/V buffered solution)-fixed and paraffin-embedded (FFPE) tumor specimens were retrieved from the BioBank of the Medical University of Graz, Austria. In 40% of the cases tumor tissue snap frozen in liquid nitrogen was available. All cancer patients were classified according to the TNM Classification of Malignant Tumors of the International Union Against Cancer (UICC) [24]. Seventeen (57%) RCC tumors were staged T1a, five (17%) were classified as T1b, four (13%) as T2a and four (13%) as T3a, respectively. Six (20%) of RCC tumors were graded G1, 16 (53%) G2 and 8 (27%) G3, respectively. In the tumors of six patients (16%) venous invasion was found.

**Table 1:** Clinical and histopathological characteristics of the study cohort

**Parameter**	**Patients, n; (%)**
**Age, median; (range)**	68; (30–83)
Men	28; (70)
Women	12; (30)
**Benign tumors**	10; (25)
Renal cysts	1; (2.5)
Angiomyolipoma	2; (5)
Oncocytoma	6; (15)
Cystic Nephroma	1; (2.5)
Tumor size (cm), median; (range)	2.5; (1.0-6.0)
**Malignant tumors**	30; (75)
Clear cell RCC	25; (62.5)
Papillary RCC	5; (12.5)
Tumor size (cm), median; (range)	4; (1.3-12.0)
**Tumor differentiation**	
G1	6; (20)
G2	16; (53.3)
G3	8; (26.7)
Venous invasion (in cases of potentially invasive tumors)	6; (16)
Distant metastasis (in cases of potentially invasive tumors)	1; (3)
**Type of surgical intervention**	
Open partial nephrectomy	24; (60)
Open nephrectomy	6; (15)
Transperitoneal nephrectomy	4; (10)
Laparoscopic nephrectomy	3; (7.5)
Laparoscopic partial nephrectomy	2; (5)
CT targeted biopsy	1; (2.5)

### Blood sample collection

From each patient a total of 4 blood samples were collected in 2 × 6 ml EDTA tubes (Greiner Bio-One, Kremsmünster, Austria) from a peripheral vein. The first sample was drawn one day prior to surgery (time point A), the next time points of collection were once, during surgery shortly after removal of the tumor (time point B), one day after surgery (time point C) and eight days after surgery (time point D).

### DNA extraction of primary tumor tissue

Genomic DNA was extracted from snap frozen tumor tissue or if not available, from FFPE tumor tissue using the QIAamp DNA Mini Kit (Qiagen, Hilden, Germany) as described by the manufacturer. DNA quantity and quality was assessed by Nanodrop measurement (Thermo Fisher Scientific, Waltham, USA) and DNA fragmentation was analysed by agarose gel electrophoresis [25].

### Cell lines and evaluation of the sensitivity of the ScreenCell® filtration device

The female renal cell adenocarcinoma cell line 769-P was purchased from ATCC (Manassas, USA) and cultivated in RPMI supplemented with 10% fetal calf serum, 1 mM sodium pyruvate, 100U/ml penicillin and 100 μg/ml streptomycin (all cell culture supplies from Gibco, Invitrogen, Carlsbad, USA). The human iliac arterial endothelial primary cells (HIAEC) (generously provided by Dr. I. Lang-Olip, Medical University of Graz, Austria) were grown in endothelial growth medium-2 (EGM-2) (Lonza, Walkersville, USA).

The sensitivity of the ScreenCell® filtration method was evaluated in nine independent experiments by spiking cultured renal cell cancer cells (769-P cell line) into blood samples from a healthy volunteer. Confluent 769-P cells were harvested by trypsinization using 0.05% Trypsin-EDTA buffer (PAA, Pasching, Austria). Then defined numbers (three batches of 2, 10 and 50 individual cells) were picked under an inverted microscope (Zeiss Axiovert M 200, Munich, Germany) using a micromanipulator (MMJ, Zeiss; CellTram vario, Eppendorf, Hamburg, Germany) equipped with microcapillaries (inner diameter of 20 μm) (TransferTip, Eppendorf). Picked cells were directly transferred into the blood sample which was then processed by filtration and hematoxylin staining. Cells were enumerated and recovery rates were calculated.

### ScreenCell® filtration

All blood samples were processed within 4 hours after collection of blood as recommended by the manufacturer (ScreenCell®, Paris, France). Filtration was carried out as previously described [15] with minor modifications. Briefly, blood was diluted 8 fold with red blood cell lysis buffer (ScreenCell®, Paris, France) and incubated for 10 min at RT, with gentle agitation after 3 and 6 min. Per patient and time point 4 filtrations, each corresponding to the processing of 2 ml of whole blood were performed using vacutainer tubes as described by the manufacturer. Thereafter the filters were rinsed with 2 ml of sterile phosphate-buffered saline (PBS pH 7.4) and collected from the device. The filters were counterstained with hematoxylin (Merck, Darmstadt, Germany) for 5 min and blued for a few seconds with NH_3_-H_2_O (0.06% m/V), washed in distilled water, air dried and mounted on a glass slide for evaluation by light microscopy. Of 14 selected cases (11 patients with RCC, 1 with oncocytoma, and 2 cases with benign tumors) three of the four filters remained unstained and were stored at −20°C until further immunocytochemical analysis.

### Cytomorphological analyses of filtered cells

**Figure 1:**
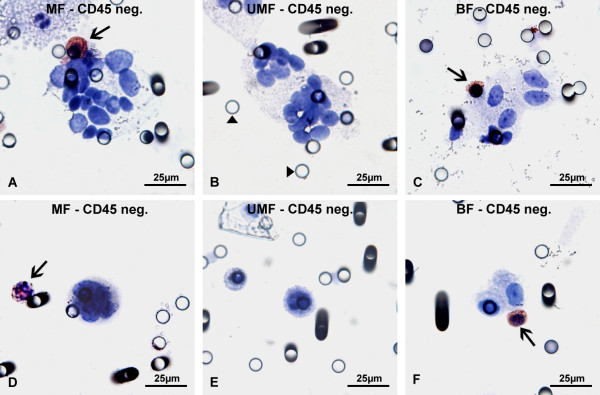
**Immunocytochemical analysis of CNHCs with antibodies against the hematological marker CD45.** Cellular aggregates (clusters) of non-hematological cells (CNHC) isolated from blood of patients with renal tumors by ScreenCell® filtration **(A**-**C)** with cytomorphological features of malignant (CNHC-MF) **(A)**, uncertain malignant (CNHC-UMF) **(B)**, and benign cells (CNHC-BF) **(C)** as previously defined [17]. None of the CNHC are detected by CD45 antibodies, whereas single lymphocytes are CD45-positive (indicated by arrows in **A** and **C**). Examples of filter pores (8 μm in diameter) are marked by arrow heads **(B)**. Single CNHC isolated by ScreenCell® filtration **(D**-**F)** with cytomorphological features of malignant (−MF) **(D)**, uncertain malignant (−UMF) **(E)**, and benign cells (−BF) **(F)** are CD45-negative. However, leucocytes are stained with CD45 antibodies (arrows in **D** and **F**).

Stained filters were analysed by light microscopy by a board certified cytopathologist (CL) using the cytomorphological criteria recently issued by a panel of ten expert cytopathologists for the classification of circulating non-hematological cells (CNHC) filtered from blood using the method “isolation by size of epithelial tumor cells” (ISET) as published by Hofman et al. [17]. This filtration technology is based on comparable principles for isolation of CTCs/CTMs from blood, i.e. size exclusion by filtration through a translucent polycarbonate membrane filter with 8 μm pore size using vacuum suction, as the ScreenCell® system. Therefore cytomorphological criteria established with ISET filtration may also be used to evaluate filtered cells on ScreenCell® filters. The proposed criteria by Hofman have also been applied to renal cell carcinoma in one study [17]. According to these criteria filtered cells >8 μm are classified as CNHC with malignant (CNHC-MF), uncertain malignant (CNHC-UMF) and benign features (CNHC-BF) (Figure 1A-F).

CNHC occurring as cellular aggregates (clusters) were classified as CNHC-MF (Figure 1A) if at least four of the following criteria were present:

○ Anisonucleosis (ratio >0.5)

○ Nuclei larger than 3 times the calibrated 8 μm pore size of the membrane

○ Irregular nuclear outline

○ Presence of tridimensional cellular sheets

○ High nuclear/cytoplasmic ratio

CNHC clusters were defined as CNHC-UMF when less than 4 of these criteria were seen (Figure 1B), whereas CNHC-BF did not show any of these characteristics (Figure 1C).

Single CNHC (Figure 1D-F) were classified as CNHC-MF (Figure 1D) if all 3 of the following cytological criteria were present:

○ Nuclei larger than 3 times the calibrated 8 μm pore size

○ Irregular nuclear outline

○ High nuclear/cytoplasmic ratio

If less than 3 of these criteria were present, the single cell was CNHC-UMF (Figure 1E). CNHC-BF cells showed none of the cellular features described above (Figure 1F). The number of cells was assessed by counting nuclei of single CNHCs and within CNHC clusters. The total numbers of CNHCs are listed in Table 2.

**Table 2:** Summary of the results of cytomorphological classification of CNHCs detected by ScreenCell® filtration in 8 ml of venous blood of patients with benign (n = 10), malignant (n = 30) renal tumors and healthy controls (n = 20)

					**Number of CNHC / 8 ml**											
		**Clinical data**			Time point A*			Time point B**			Time point C***			Time point D****		
**Patient number**	**Histological diagnosis**	**TNM**	**Grade**	**Venous invasion**	**MF**	**UMF**	**BF**	**MF**	**UMF**	**BF**	**MF**	**UMF**	**BF**	**MF**	**UMF**	**BF**
26	angiomyolipoma	-	-	no	0	2	0	0	0	0	0	0	0	0	0	0
33	angiomyolipoma	-	-	no	0	0	0	0	0	0	0	0	0	0	0	0
29	benign cyst	-	-	-	0	0	0	0	10	0	0	0	30	2	0	0
18	cyst. nephroma	-	-	-	51	4	0	2	0	0	0	127	30	30	380	0
3	oncocytoma	-	-	no	0	560	2	0	0	0	0	0	0	0	251	195
4	oncocytoma	-	-	no	0	0	0	0	2	0	0	40	8	0	2	3
6	oncocytoma	-	-	no	0	3	0	0	0	0	2	35	35	0	19	11
14	oncocytoma	-	-	no	0	0	0	0	39	0	37	8	0	21	5	0
17	oncocytoma	-	-	no	2	136	0	0	0	0	1	0	0	2	0	35
38	oncocytoma	-	-	no	0	0	4	0	0	0	0	0	0	0	0	0
1	ccRCC	T2aN0M0	2	yes	0	0	0	0	0	0	ND	ND	ND	ND	ND	ND
2	ccRCC	T3aN0M0	2	yes	0	0	0	0	0	3	0	10	0	0	0	0
7	ccRCC	T2aN0M0	2	no	0	0	0	0	0	0	0	6	0	ND	ND	ND
9	ccRCC	T1bN0M0	2	no	3	19	5	0	0	0	ND	ND	ND	0	0	0
10	ccRCC	T1N0M0	2	no	16	0	10	0	0	0	0	0	3	0	22	16
11	ccRCC	T1N0M0	2	no	0	0	0	2	0	0	ND	ND	ND	0	0	0
13	ccRCC	T3aN0M0	3	yes	0	19	0	0	0	0	22	158	30	ND	ND	ND
15	ccRCC	T1aN0M0	2	no	0	0	0	0	0	0	0	0	0	0	0	0
16	ccRCC	T1aN0M0	2	no	62	56	0	0	0	0	0	0	0	0	0	0
19	ccRCC	T1aN0M0	1	no	1	0	0	0	0	0	0	0	0	0	3	5
20	ccRCC	T3aN0M0	3	yes	13	5	0	0	0	0	20	319	58	ND	ND	ND
22	ccRCC	T1aN0M0	1	no	0	32	0	1	0	0	11	0	0	ND	ND	ND
23	ccRCC	T1aN0M0	1	no	1	0	0	0	1	0	2	0	10	5	12	15
24	ccRCC	T1N0M0	1	no	ND	ND	ND	0	0	5	0	1	0	0	0	0
25	ccRCC	T1aN0M0	2	no	0	1	0	0	0	0	0	1	20	ND	ND	ND
28	ccRCC	T1bN0M0	1	no	0	0	0	0	645	0	5	5	25	0	36	12
30	ccRCC	T1N0M0	3	no	0	0	0	31	227	4	0	0	0	0	67	64
32	ccRCC	T1aN0M0	2	no	1	0	0	1	0	0	3	26	15	ND	ND	ND
34	ccRCC	T1aN0M0	2	no	0	0	0	0	26	51	0	18	0	ND	ND	ND
35	ccRCC	T1aN0M0	2	no	0	0	0	0	0	0	ND	ND	ND	0	20	0
36	ccRCC	T1aN0M0	3	no	2	125	3	0	0	0	2	1	7	0	0	0
40	ccRCC	T1aN0M0	3	no	0	23	0	0	0	0	0	0	15	0	0	0
41	ccRCC	T1aN0M0	3	no	0	0	0	0	0	0	1	0	0	3	74	4
42	ccRCC	T1N0M0	1	no	0	0	0	0	0	0	0	22	44	58	124	98
27	pap.RCC	T1bN0M0	3	no	0	0	0	0	0	0	0	0	0	1	6	0
8	pap.RCC	T1N0M0	2	no	ND	ND	ND	0	0	0	0	0	0	0	0	0
12	pap.RCC	T3aN0M0	3	yes	0	0	5	0	0	0	11	0	0	ND	ND	ND
31	pap.RCC	T1aN0M0	2	no	0	0	0	0	0	0	ND	ND	ND	ND	ND	ND
37	pap.RCC	T2aN0M0	2	no	0	0	100	19	79	18	1	0	4	ND	ND	ND
39	reg RCC	T2aN0M0	2	yes	0	0	1	1	0	0	1	0	0	1	0	0
C1	Control	-	-	-	0	0	1	-	-	-	-	-	-	-	-	-
C2	Control	-	-	-	0	0	0	-	-	-	-	-	-	-	-	-
C3	Control	-	-	-	0	2	2	-	-	-	-	-	-	-	-	-
C4	Control	-	-	-	0	83	100	-	-	-	-	-	-	-	-	-
C5	Control	-	-	-	0	0	0	-	-	-	-	-	-	-	-	-
C6	Control	-	-	-	0	0	0	-	-	-	-	-	-	-	-	-
C7	Control	-	-	-	0	0	12	-	-	-	-	-	-	-	-	-
C8	Control	-	-	-	0	0	0	-	-	-	-	-	-	-	-	-
C9	Control	-	-	-	0	1	0	-	-	-	-	-	-	-	-	-
C10	Control	-	-	-	0	0	0	-	-	-	-	-	-	-	-	-
C11	Control	-	-	-	0	1	1	-	-	-	-	-	-	-	-	-
C12	Control	-	-	-	0	0	0	-	-	-	-	-	-	-	-	-
C13	Control	-	-	-	0	1	2	-	-	-	-	-	-	-	-	-
C14	Control	-	-	-	0	0	0	-	-	-	-	-	-	-	-	-
C15	Control	-	-	-	0	0	0	-	-	-	-	-	-	-	-	-
C16	Control	-	-	-	0	27	5	-	-	-	-	-	-	-	-	-
C17	Control	-	-	-	0	1	7	-	-	-	-	-	-	-	-	-
C18	Control	-	-	-	0	0	0	-	-	-	-	-	-	-	-	-
C19	Control	-	-	-	0	0	0	-	-	-	-	-	-	-	-	-
C20	Control	-	-	-	0	1	0	-	-	-	-	-	-	-	-	-

* One day before surgery.

** During surgery, after the removal of the tumor.

*** One day after surgery.

**** Eight days after surgery.

ND Not done (sample not available).

### Immunocytochemical characterization of filtered cells

After filtration the filters were dissembled from the device, air-dried over night at room temperature (RT) and then fixed in 4% m/V formaldehyde (Labonord, Templemars, France) for 10 min. After rehydration in Tris-buffered saline (TBS) for 10 min, the filters were incubated in TBS-Triton (TBS containing 0.2% Triton X-100) (Merck, Darmstadt, Germany) for 5 min. Following a washing step with TBS, hydrogen peroxidase blocking solution (Dako, Glostrup, Denmark) was applied for 10 min followed by incubation with Ultra V Block (Dako, Glostrup, Denmark) at RT for 5 min. To circumvent the effect of a possible EMT-associated low expression of epithelial antigens like cytokeratins in CTCs/CTMs and because renal cell cancers only weakly express epithelial cell adhesion molecule (EpCAM) [26], carbonic anhydrase IX (CAIX) was used as a marker. This protein is overexpressed in 100% of clear cell renal cell carcinomas and 57% of papillary renal cell carcinomas [27] but is not expressed in normal renal tissue [28]. The filters were incubated with primary antibodies directed against CAIX (rabbit IgG, NB100-417, Novus Biologicals, Littleton, USA; 3.5 μg/ml ), CD31 (mouse IgG1, M0823, Dako, Glostrup, Denmark; 1 μg/ml) or CD45 (mouse IgG1, M0855, Dako, Glostrup, Denmark; 2.4 μg/ml) diluted in Antibody Diluent (Dako, Glostrup, Denmark) for 30 min at RT followed by application of primary antibody enhancer (Dako, Glostrup, Denmark) for 15 min. Thereafter the filters were incubated with labelled polymer-HRP Anti-Mouse + Anti-Rabbit (Dako, Glostrup, Denmark) for 10 min. AEC substrate chromogen (Dako, Glostrup, Denmark) was applied for 10 min and rinsed off with distilled water prior to counterstaining with hematoxylin (Merck, Darmstadt, Germany) as described above. Respective non-immune rabbit IgG and isotype-matched monoclonal antibodies were used as controls. Cultured 769-P and HIAEC cells were spiked in blood, filtered and stained like patient samples and served as positive or negative control for staining, enumeration and array-CGH experiments.

### Laser capture microdissection and whole genome amplification of selected filtered cells

**Figure 2:**
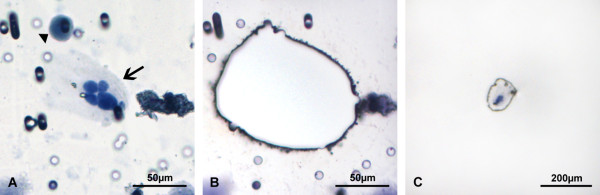
**Laser capture microdissection of a CNHC cluster.** Filter membrane with a single CNHC (arrow head) and a CNHC cluster (arrow) **(A)**. The single CNHC was removed by laser ablation before the membrane surrounding the cluster was cut by laser beam. The membrane and CNHC cluster was then laser pressure catapulted into the lid of a reaction tube **(B**-**C)**.

Isolation of selected CNHCs was carried out by laser capture microdissection (P.A.L.M., Zeiss, Munich, Germany). Before laser capture microdissection, all non-target cells and cellular debris were removed by laser ablation. The polycarbonate filter was then cut with a pulsed laser beam and the membrane and target cells were catapulted into the lid of a 200 μl PCR tube, which contained 10 μl of WGA-4 fragmentation and lysis buffer (#WGA4, Sigma-Aldrich, St. Louis, USA) (Figure 2). An exemplary laser capturing is shown in Additional file 1: Movie 1. For control purposes neutrophilic granulocytes, which were subjected to the same filtration and staining procedures like patient samples, were as well isolated by laser capture microdissection and forwarded to whole genome amplification (WGA). WGA was performed as previously described [29] with minor modifications using the GenomePlex Single Cell Whole Genome Amplification Kit (Sigma-Aldrich, St. Louis, USA). Briefly, the captured cells were digested in 10 μl fragmentation and lysis buffer, followed by GenomePlex library preparation. Amplification was performed by adding 7.5 μl of 10× amplification master mix, 48.5 μl nuclease free water and 5 μl WGA DNA polymerase. After whole genome amplification, DNA was purified using the GenElute PCR Clean-Up Kit (Sigma-Aldrich, St. Louis, USA). DNA concentration and purity was determined by Nanodrop spectrophotometer (Thermo Fisher Scientific, Waltham, USA). DNA quality was assessed by multiplex PCR as previously described [25]. Samples showing three to four DNA bands between 100 and 400 bp were regarded as of high DNA quality.

### Array-CGH

Array-CGH was performed using SurePrint G3 Human CGH Microarrays 8×60K (Agilent Technologies, Santa Clara, USA). Prior to labelling, genomic DNA derived from snap frozen and FFPE tissues as well as the corresponding reference DNA were digested by Alu I and Rsa I (Promega, Fitchburg, USA) according to the supplier’s instructions. Amplified DNA of CNHCs and the corresponding amplified reference DNA (Promega, Fitchburg, USA) showed mean DNA size distributions of 400 - 600 bp and therefore provided suitable templates for array-CGH without any digestion step. The purified samples were labelled with the Bioprime Array CGH Genomic Labeling System (Life Technologies, Carlsbad, USA). Briefly, 250 ng of sample DNA and 250 ng of female or male reference DNA (Promega, Fitchburg, USA) were labelled with dCTP-Cy5 and dCTP-Cy3 (GE Healthcare, Little Chalfont, UK), respectively. Subsequently, DNA was purified with Amicon Ultracel-30 filters (Millipore, Billerica, USA) and mixed in equal amounts. The mixture was hybridized to a 60 K array ON using the Oligo aCGH/ChIP-on-chip Hybridization kit (Agilent Technologies, Santa Clara, USA). Following hybridization, the array was washed and scanned (Agilent Technologies, Santa Clara, USA) as recommended by the manufacturer. Data analysis was performed with Agilent Genomic Workbench Lite Edition 6.5.0.18. (Agilent Technologies, Santa Clara, USA). The following settings were used for tumor tissues: ADM-2, threshold 8.0, with at least 10 consecutive oligos with an absolute log ratio of 0.22. For amplified DNA the algorithm ADM-2, threshold 9.3 was used with at least 100 consecutive oligos with an absolute log ratio of 0.30.

### Definition of thresholds and controls for array-CGH

**Figure 3:**
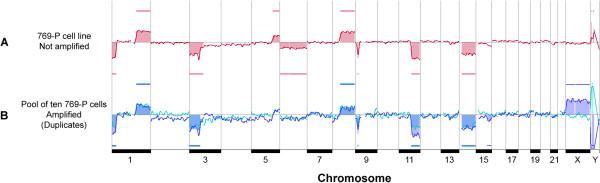
**Control array-CGH profiles of the renal cancer cell line 769-P.** DNA of the non-amplified 769-P cell line reveals gains of chromosomes 1q, 5q, 8q and losses of 1p, 3p, 6, 9p, 11q, 14 (**A**, red profile). The corresponding array-CGH profiles of amplified DNA of two biological replicates (ten 769-P cells each) show concordant gains of 1q, 8q, losses of 1p, 3p, 9p, 11q, 14 (**B**, blue and green profile, respectively). In one of the 769-P cell pools there was an additional loss of 15q (**B**, blue profile). Gains and losses of the X- and Y-chromosomes (blue profile in **B**) do not reflect true copy number variations. They result from differences between the sex of the reference and the samples DNA (e.g. female sample DNA was hybridized against a male reference DNA thereby resulting in a gain of X and loss of Y chromosome).

As it was our purpose to characterize CNHC clusters, pools of leucocytes and 769-P cells were used to test the detection limits of genetic aberrations of the array-CGH method. 769-P cells and leucocytes were subjected to ScreenCell® filtration, microdissected from the filters, the DNA was subjected to WGA and further analysed by array-CGH. There was concordance of gains and losses of chromosomal DNA detected in amplified pools of ten 769-P cells and non-amplified DNA from cultured 769-P cells [30] (Figure 3). From this comparison we estimated that deletions of approximately ~6.8 Mb can be detected in pools of ten 769-P cells (chromosome 9, genomic position 16.2-23.0 Mb) which is sufficient to indicate larger scale aberrations in RCC [31]*.* The DNA of isolated pools of 10 leucocytes from blood of a healthy individual, representing a balanced genome, was used to set the thresholds for the detection limits of gains and losses by array-CGH in our study. In contrast to cell cultured cells, the array-CGH profiles of amplified DNA of CNHCs demonstrated slightly noisier ratio profiles, as we expected if going from an artificial cell culture system to clinical samples. By applying the above mentioned threshold settings, gains and losses could be reliably detected (Figure 3).

### Statistical analysis

We investigated if the presence or absence of CNHC types (categorized as binary variables) differed between time points A–D, using Chi-square tests. Furthermore, median, minimum and maximum were used to describe the number of CNHCs of each type and for every time point. The associations between numbers of CNHCs of each type with tumor size, venous invasion and differentiation grade were explored using nonparametric methods. A p-value of <0.05 was considered to indicate statistical significance. All p-values were regarded in an explorative sense. The statistical calculations were performed using the SPSS software package, version 20.0 (IBM, Armonk, USA).

## Results

### Spiking experiments

The average number of recovered 769-P cells for 50, 10 and 2 spiked cells were 45.3 (SD 2.1), 8.7 (SD 1.5), and 1.7 (SD 0.6), respectively. The average recovery rates of 769-P were 91%, 87% and 83% for 50, 10 and 2 spiked 769-P cells, respectively which compares to sensitivity rates published by Desitter et al. for the ScreenCell® filtration device [15].

### Cytomorphological analysis of CNHC types in patients with renal tumors and healthy controls

**Figure 4:**
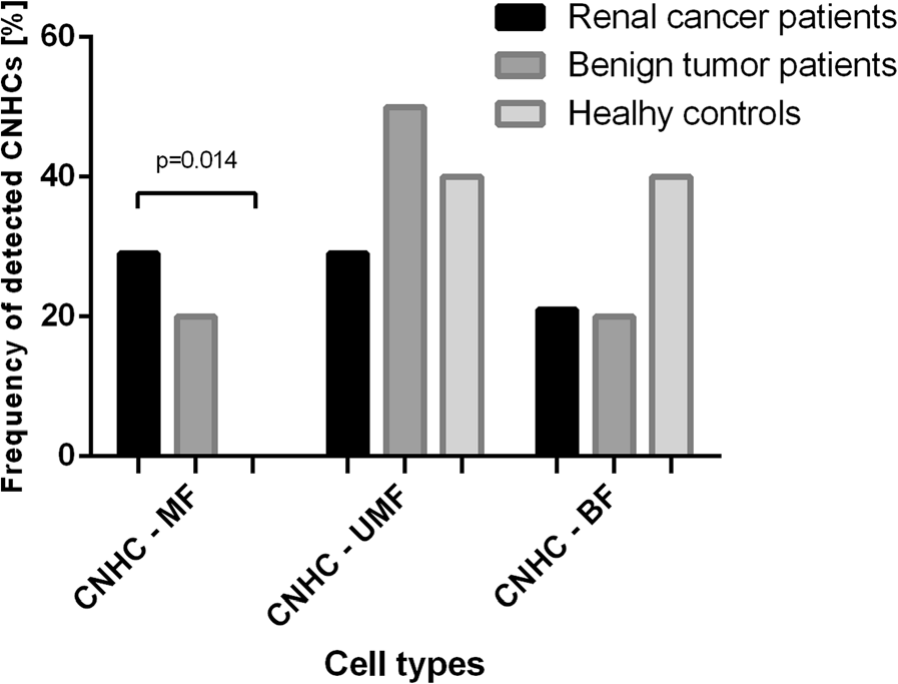
**Overall frequencies of the CNHC types in healthy controls and patients before surgery.** Percentage of blood samples positive for CNHC-MF, -UMF and BF- types. In renal cancer patients, CNHC of the MF-type were significantly more frequently detected than in healthy controls (29% vs 0%, p = 0.014).

Overall CNHCs of the MF-type were detected more frequently in renal cancer patients (29%) as compared to healthy controls (0%) (p = 0.014). CNHC-MF were also found in 20% of cases with benign renal tumors which was not significantly different from the frequency found in healthy controls (p = 0.103). However in healthy controls CNHC-MF were not found. CNHC-UMF and –BF types were identified in the blood of 29% and 21% of the renal cancer patients, in 50% and 20% of benign renal tumor patients as well as in 40% and 40% of healthy individuals (Figure 4).

A summary of the results of the cytomorphological analyses and the number of cells of all patients and time points is provided in Table 2.

**Figure 5:**
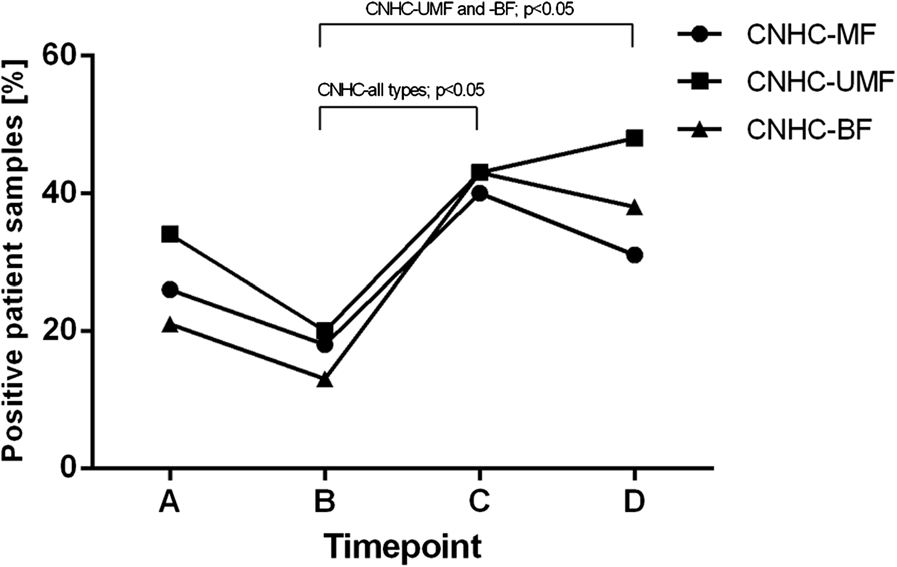
**Percentage of patient samples positive for CNHC-MF, -UMF and BF at different time points of sampling.** Percentage of blood samples positive for CNHC-MF (circle), CNHC-UMF (rectangle), and CNHC-BF (triangle) one day before surgery (time point **A**), during surgery, after the removal of the tumor (time point **B**), one day after surgery (time point **C**), and 8 days after surgery (time point **D**). There was no significant change in the percentage of blood samples positive for CNHC of any type after surgery (time points **C** and **D**, respectively) as compared to before surgery (time point **A**). However, a significant increase of samples positive for CNHCs of every type was found at time point **C** as compared to time point **B** (p < 0.05, each). Blood samples positive for CNHC-UMF and CNHC-BF but not CNHC-MF, were also more frequently detected at time point **D** as compared to time point **B**.

Overall, one day before surgery (time point A) 26%, 34%, and 21% of the blood samples were positive for CNHC-MF, -UMF and –BF, respectively. However, during surgery, after removal of the renal tumor (time point B) CNHC-MF, -UMF and -BF were found in only 18%, 20% and 13% of samples, respectively. One day after surgery (time point C) there was a significant increase in the number of blood samples positive for CNHC-MF (40%, p = 0.040), -UMF (43%, p = 0.045) and BF (43%, 0.004). Eight days after surgery (time point D) 48% of the samples were positive for CNHC-UMF and 38% were positive for CNHC-BF, whereas in only 31% of samples CNHC-MF were found (Figure 5).

The cytomorphological CNHC types, CNHC-MF,-UMF,-BF either as single cells or clusters were found at every time point in patients regardless of the histological tumor diagnosis (Table 2).

**Figure 6:**
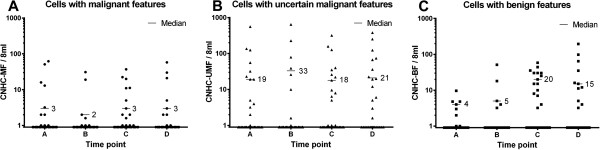
**Numbers of CNHC-types at different time points.** Numbers of CNHC-MF **(A)**, -UMF **(B)**, and –BF **(C)** per 8 ml of venous blood at time points **A**-**D**. Median cell numbers are given for each time point.

The median number of CNHC-MF per 8 ml of blood was 3 cells (range: 1–62) before surgery (time point A), 2 cells (range: 1–31) during the surgery (time point B), 3 cells (range: 1–37) one day after surgery (time point C) as well as 3 cells (range: 1–58) eight days after surgery (time point D) (Figure 6A). The median number of CNHC-UMF per 8 ml of blood was 19 cells (range: 1–560) at time point A, 33 cells/8 ml (range: 1–645) at time point B, 18 cells/8 ml (range: 1–319) at time point C and 21 cells/8 ml (range 2–380) at time point D (Figure 6B). Cells with benign features represented with a median number of 4 cells/8 ml (range: 1–100) at time point A, 5 cells/8 ml (range: 3–51) at time point B, 20 cells/8 ml (range: 3–58) at time point C and 15 cells/8 ml (range: 3–195) at time point D (Figure 6C). In renal cancer patients there was no correlation between histopathological tumor parameters (tumor size, grade of differentiation) and detection of CNHC regardless of type or time point examined (Additional file 2: Table S1 and Additional file 3: Table S2). However, one day after surgery (time point C) higher numbers of CNHC-MF were detected in patients with RCC with venous invasion as compared to those cases without venous invasion (p = 0.013). This was not found at any of the other time points investigated (Additional file 4: Table S3).

In the blood of healthy controls no CNHC-MF were detected. In three out of 20 cases (15%) we found CNHC clusters, primarily of the BF- (14 clusters) and UMF-types (6 clusters). In 7 out of 20 cases (35%) we found single CNHCs, mostly of the BF- (13 cells) and less frequently the UMF-types (5 cells). Overall, 0%, 40%, and 40% of the blood samples from healthy controls were positive for CNHC-MF, -UMF and -BF, respectively (Table 2). All detected CNHCs were negative for CAIX or CD45. However, 4 CNHC clusters of the –BF type and 2 of the –UMF type were positive for CD31.

### Immunocytochemical analysis of CNHC in patients with renal tumors

Results of the immunocytochemical analyses are compiled in Table 3.

**Table 3:** Summary of the immunocytochemical analysis of CNHCs with antibodies against CD45, CD31, and CAIX

	***Single *CNHCs positive with CD45/CAIX/CD31 antibodies**			**CNHC *clusters *positive with CD45/CAIX/CD31 antibodies**		
	**N (%)**			**N (%)**		
	**CD45**	**CAIX**	**CD31**	**CD45**	**CAIX**	**CD31**
**CNHC-MF**	0/3 (0%)	5/9 (56%)	0/1 (0%)	0/5 (0%)	None detected	3/3 (100%)
**CNHC-UMF**	0/4 (0%)	0/4 (0%)	1/3 (33%)	0/12 (0%)	1/16 (6%)	17/17 (100%)
**CNHC-BF**	0/2 (0%)	0/1 (0%)	0/6 (0%)	0/8 (0%)	0/12 (0%)	8/13 (62%)

On the 53 filters stained with antibodies against CD45 (Figure 1A-F), hematological cells including lymphocytes and polymorphic nuclear granulocytes showed a positive staining, whereas the CNHC-MF, -UMF and –BF, either present as cell clusters (Figure 1A-C) or as single cells (Figure 1D-F) were without exception negative.

**Figure 7:**
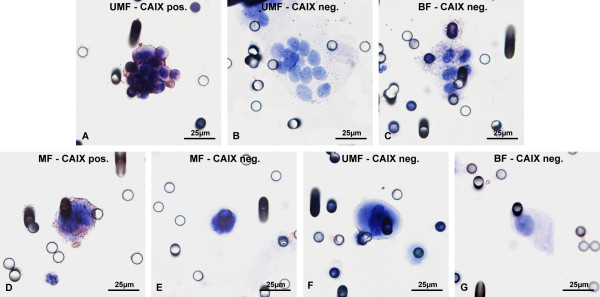
**Immunocytochemical analysis of CNHCs with antibodies against the RCC marker CAIX.** Clusters of CNHCs cytomorphologically classified as uncertain malignant (−UMF) with cytoplasmic positive staining with antibodies against the RCC marker CAIX **(A)**. Clusters of CNHC-UMF and -BF without reactivity for CAIX antibodies (**B** and **C**, respectively). A single CNHC-MF with positive cytoplasmic **(D)** and without staining for CAIX **(E)**. Single CAIX-negative CNHC-UMF and -BF (**F** and **G**, respectively).

On the 46 filters stained with antibodies against the RCC marker CAIX (Figure 7), 28 CNHC clusters were detected. None exhibited cytological features of malignancy, 16 were classified as CNHC-UMF and 12 were CNHC-BF. Of the 16 CNHC-UMF clusters, one (6%) was decorated by CAIX antibodies (Figure 7A), 15 (94%) were negative (Figure 7B), as was the case with the 12 CNHC-BF (Figure 7C). Nine single CNHC-MF were detected, 5 (56%) of which exhibited positive cytoplasmic reactivity (Figure 7D), whereas 4 (44%) were negative (Figure 7E). Only 4 single CNHC-UMF and one CNHC-BF were found and these cells were also CAIX-negative (Figure 7F and 7G, respectively).

**Figure 8:**
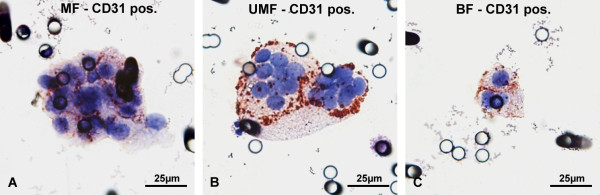
**Immunocytochemical analysis of CNHCs with antibodies against the endothelial marker CD31.** Clusters of CNHC-MF **(A)**, -UMF **(B)**, and -BF **(C)**, with cytoplasmic staining for CD31.

Immunocytochemical analysis of 14 filters with antibodies against the endothelial cell marker CD31 (Figure 8) yielded 3 CNHC-MF- and 17 CNHC-UMF cell clusters, all of which were CD31 positive (Figure 8A and B, respectively), as were 8 (62%) of the 13 CNHC-BF clusters (Figure 8C). Of single cells, only one cell with UMF was CD31 positive.

### Array-CGH

**Figure 9:**
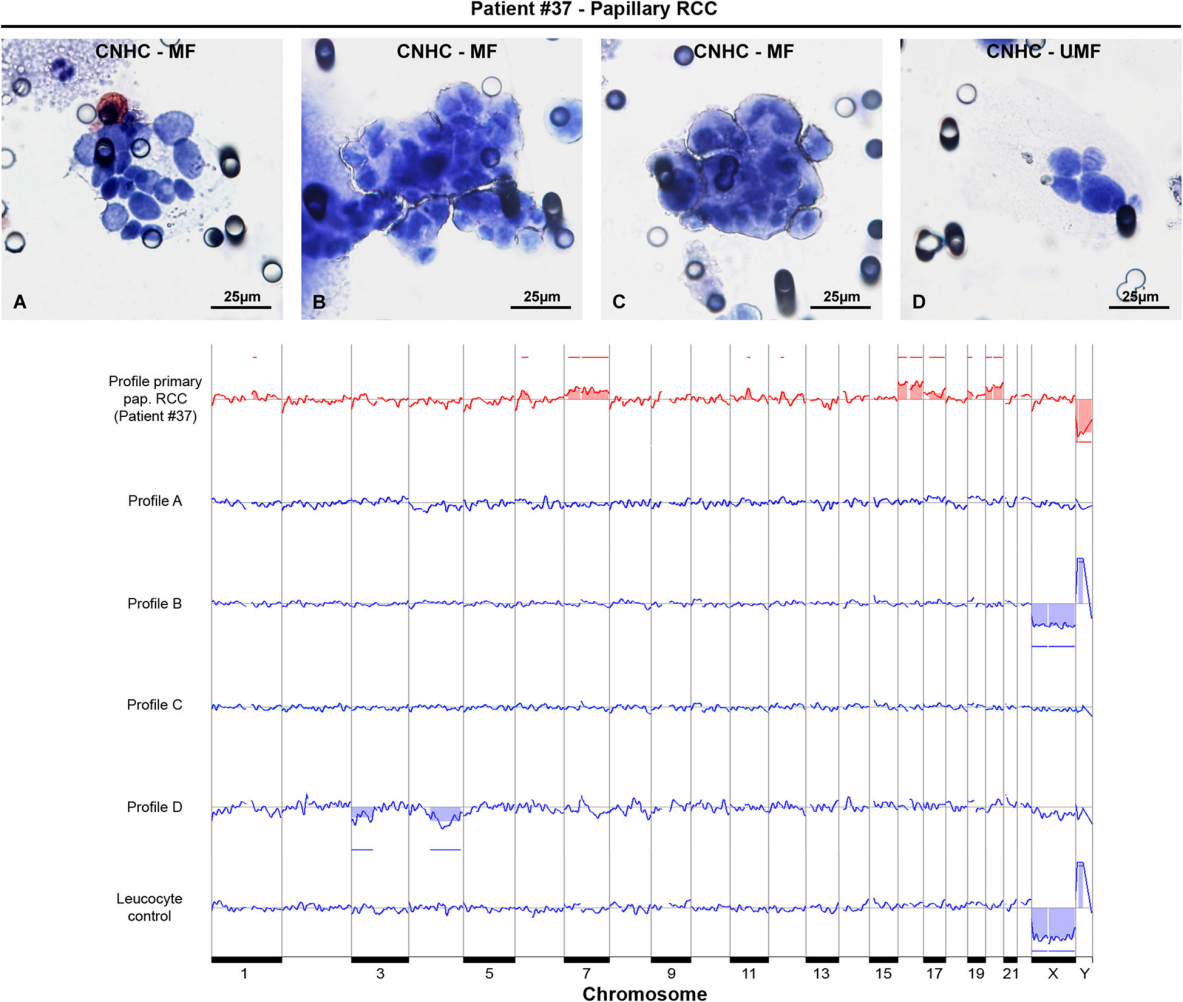
**Array-CGH profiles of the DNA of a papillary RCC and the respective CNHCs.** The DNA of the papillary RCC of patient #37 shows gains at chromosomes 6p, 7, 16, 17q, 20 and loss of chromosome Y, changes typically found in pap. RCC (red profile). However, the array-CGH profiles of three CNHC-MF clusters (photographs **A-C**) are balanced (profiles **A-C**), whereas losses at chromosomes 3p and 4q are identified in the DNA of a CNHC-UMF cluster (photograph **D** and profile **D**, respectively) isolated from the same patient. The two clusters shown in photograph **A** and **D** are negative for CD45. All other clusters were hematoxylin stained. As a control, the DNA of an isolated pool of 10 leucocytes from blood of a healthy individual showed a balanced genome (leucocyte control). Gains and losses of the X- and Y-chromosomes (profile **B** and leucocyte control) do not reflect true copy number variations. They result from differences between the sex of the reference and the samples DNA (i.e. male patient DNA was hybridized against a female reference DNA thereby resulting in a loss of X and gain of Y chromosome). Bars above the x-axis are considered to be gains, below the x-axis losses of DNA.

**Figure 10:**
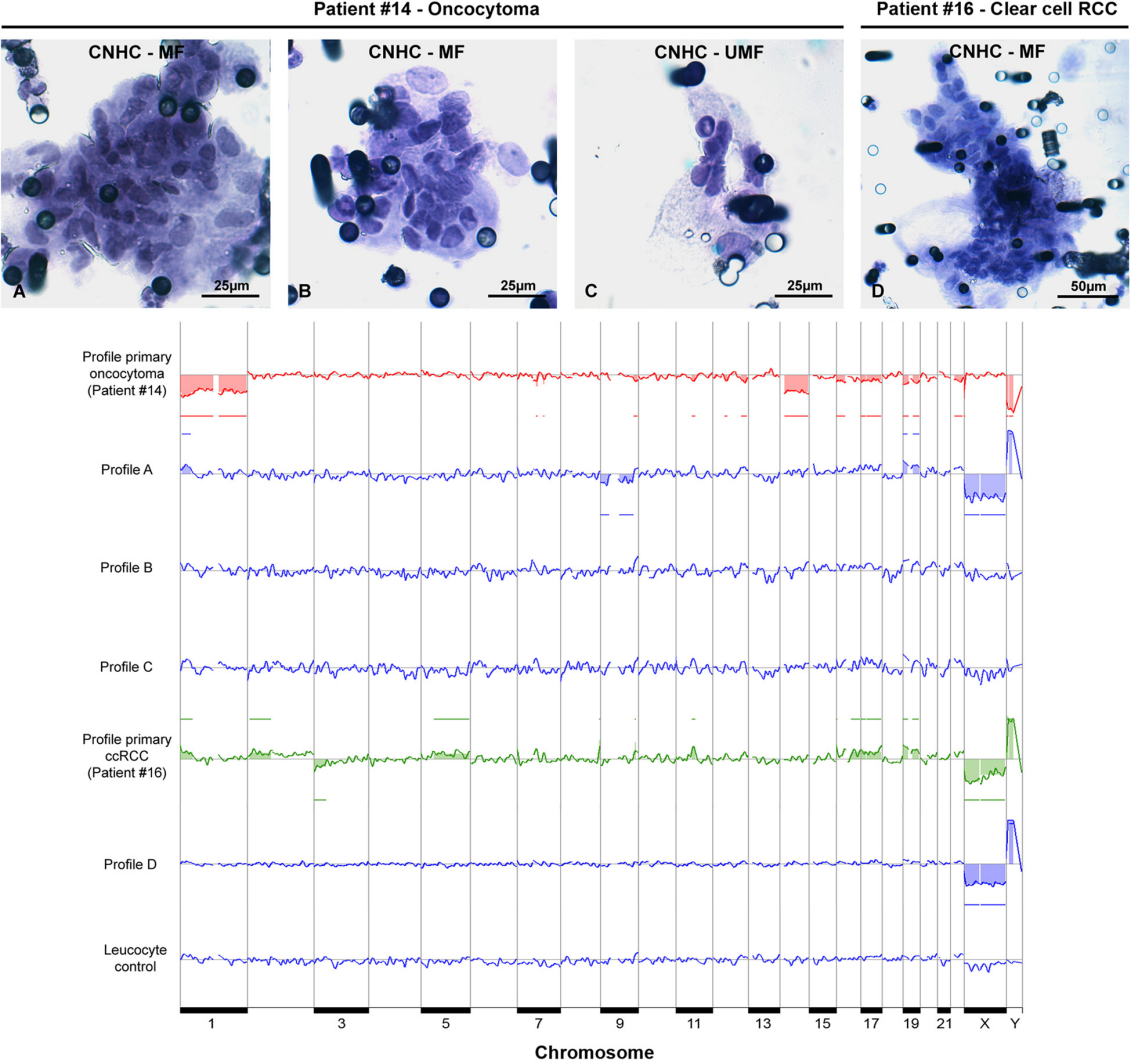
**Array-CGH profiles of the DNA of an oncocytoma or clear cell RCC and the respective CNHCs.** The DNA of the oncocytoma represents with typical losses at chromosomes 1, 14, 17, 22 and Y (red profile). Array-CGH profile of the DNA of a CNHC-MF cluster (photograph **A**) reveals a gain of 1p and loss of chromosome 9 (profile **A**), whereas the profiles of another CNHC-MF cluster (photograph **B**) and a CNHC-UMF cluster (photograph **C**) indicated no detectable copy number variations (profiles **B** and **C**, respectively). The DNA of the clear cell RCC of patient #16 reveals gains of 1p, 2p, 5q, 17 and a loss of 3p (green profile) commonly observed in clear cell RCC. The array-CGH profile of the DNA of a CNHC-MF (photograph **D**) of patient #16 is balanced (profile **D**). All clusters were hematoxylin stained. As a control, the DNA of an isolated pool of 10 leucocytes from blood of a healthy individual showed a balanced genome (leucocyte control). Gains and losses of the X- and Y-chromosomes (green profile, profile **A** and **D**) do not reflect true copy number variations. They result from differences between the sex of the reference and the samples DNA (i.e. male patient DNA was hybridized against a female reference DNA thereby resulting in a loss of X and gain of Y chromosome). Bars above the x-axis are considered to be gains, below the x-axis losses of DNA.

After WGA, 55 out of 115 (48%) stained microdissected cells yielded DNA products. According to the multiplex PCR, the DNA quality of 49% (27 of 55) of these cells was suitable for array-CGH. Six of CNHC-MF- and 6 of CNHC-UMF-types were selected for array-CGH analysis. All passed multiplex quality control and yielded informative array-CGH profiles. The DNA of the CNHC clusters and the respective renal tumor tissues, one case of papillary RCC (Figure 9), two cases of clear cell RCC (Figure 10 and Additional file 5: Figure S1), one oncocytoma (Figure 10) as well as one cystic nephroma (Additional file 5: Figure S1) were subjected to array-CGH analysis. The results are summarized in Table 4.

**Table 4:** Summary of the results of array-CGH profiling of the DNA of primary tumors and respective CNHCs

		**Chromosomal aberrations identified by array-CGH**	
**Patient number**	**Items analysed by array-CGH**	**Gains**	**Losses**
**14**	Oncocytoma	None detected	1, 14, 17, 22, Y
	CNHC-MF (A)	1p	9
	CNHC-MF (B)	Balanced	Balanced
	CNHF-UMF (C)	Balanced	Balanced
**16**	Clear cell RCC	1p, 2p, 5q, 17	3p
	CNHC-MF (D)	Balanced	Balanced
**18**	Cystic nephroma	Not analysed*	Not analysed*
	CNHC-UMF (C)**	Balanced	Balanced
	CNHC-UMF (D)**	Balanced	Balanced
**30**	Clear cell RCC**	5q, 7	3p
	CNHC-UMF (A)**	Balanced	Balanced
	CNHC-UMF (B)**	Balanced	Balanced
**37**	Papillary RCC	6p, 7, 16, 17q, 20	Y
	CNHC-MF (A)	Balanced	Balanced
	CNHC-MF (B)	Balanced	Balanced
	CNHC-MF (C)	Balanced	Balanced
	CNHC-UMF (D)	None detected	3p, 4q

* Unfortunately the quality of the DNA generated from the tumor tissue was not suitable for array-CGH due to insufficient quality.

** Array-CGH profiles are available as additional information (Additional file 5: Figure S1).

The chromosomal aberrations found in the DNA of the tumor tissues are in keeping with the genetic alterations reported for ccRCC, papRCC, and oncocytoma in the literature [31-33] and copy number aberration database (http://www.progenetix.net) [34]. DNA generated from a benign renal cyst of one patient (patient # 18) was of insufficient quality for analysis by array-CGH. Irrespective of the cytomorphological type, most of the CNHC clusters showed a balanced genome. In only two of the 12 (17%) CNHC clusters, one of which was a MF- and the other one was a UMF- cytomorphological type chromosomal aberrations could be identified which did not match the patterns of chromosomal changes found in the respective renal tumors (Table 4).

## Discussion

We evaluated the feasibility and utility of the ScreenCell® filtration system for the detection of CTCs and CTMs in the blood of patients with RCC. We investigated if morphological features can be used to discriminate between malignant and non-malignant cells by applying array-CGH.

We found the ScreenCell® filtration an easy to perform procedure which allowed for the detection of large cells (i.e. diameter of > 8 μm) after filtration of 8 ml of diluted blood in 60% of patients with benign and in 53% with malignant renal tumors one day before surgical intervention. According to their cytomorphological features, these cells either occurring as single cells or cellular clusters consisting of at least 3 cells were classified as CNHC-MF, -UMF or -BF using diagnostic criteria recently published by a panel of expert cytopathologists [17]. Surprisingly, each type of CNHCs was found in blood of patients with benign renal tumors including renal cysts, cystic nephroma, angiomyolipomas, oncocytomas as well as malignant papillary or clear cell RCCs. The presence of CNHCs did not correlate with histopathological features of the respective tumors including tumor size, histological diagnosis and grade of tumor differentiation. However, in patients with renal cancer a correlation between the CNHC-MF numbers and histological venous invasion was found at time point C (one day after surgery). At time point A (one day before surgery) CNHC-MF were also more frequently detected in renal cancer patients as compared to healthy controls.

Immunocytochemical analysis revealed that single cells or cellular clusters found on the filters could be regarded as CNHCs because they were invariably negative for CD45, an established marker for hematological cells [35]. Indeed, the results of the CD31 immunocytochemical staining and genetic analysis seem to indicate that most of the cellular CNHC clusters identified in our study may represent aggregations of circulating endothelial cells (CECs) rather than CTMs. More than half (62%) of the clusters of CNHCs-BF-type and all of the CNHC of the -MF and -UMF types found on the filters analysed with antibodies against CD31 were positive, whereas only 6% of the CNHC-UMF- and none of the CNHC-BF clusters were positive on the filters stained for the RCC marker CAIX [36,37]. Although with lower staining intensity as compared to endothelial cells, neutrophilic granulocytes, some lymphocytes, monocytes and platelets can also be stained by the CD31 antibodies [38]. Therefore we cannot exclude that the CNHC clusters may contain some of these cell types as well. However, this is not supported by their cytomorphological features and negative staining results with CD45-antibodies.

In contrast to what was observed for the CNHC clusters, 56% of the single CNHC-MF on the filters analysed with antibodies against the renal cancer marker CAIX [36,37] were positive and might thus represent “true” CTCs. Only 1 out of 10 single CNHC (10%) was positive for CD31. Unfortunately, only DNA of insufficient quality could be generated from single CNHCs of any type precluding array-CGH characterisation of these cells.

Analysis of limited amounts of DNA, as in the analysis of CTCs is faced with several technical problems. Procedures of fixation and staining can decrease DNA quality and interfere with the whole genome amplification (WGA) process [39]. Several WGA methods are available to generate sufficient quantities of DNA for array-CGH, all of which are prone to amplification bias [40-42]. We therefore used the GenomePlex library technology which has been shown to exhibit no nucleotide related amplification bias [43,44]. In addition, the reference DNA was also amplified using GenomePlex library technology to further minimize the amplification bias [45]. Array-CGH profiles from amplified DNA of few cells tend to be “noisier” than compared to non-amplified DNA [39,43]. Although higher resolutions are reported with dense array platforms [43], copy number variations as small as 6.8 Mb could be identified in our study which seems appropriate to detect large scale aberrations described in RCC [31].

CTMs have been described in several CTC isolation procedures [19,46,47], in particular with techniques relying on size filtration [11,17,20]. Based on their cytological features some were designated “atypical”, “uncertain malignant” or “morphologically doubtful” [17,48,49]. However, endothelial cells, megakaryocytes as well as large monocytes may be difficult to distinguish from “true” CTC or CTM [17]. The reason why circulating benign cells display cytological atypia is not known. Reactive changes might be introduced by shear forces in circulation and/or during filtration.

In some studies the ScreenCell® filtration technique was applied to isolate CTC/CTM from the blood of patients with adrenocortical, prostate, colon and breast cancer or malignant melanoma [15,50,51]. These cells have been characterized by immunocytochemistry or telomeric analysis. However, array-CGH data or results from immunocytochemical testing with CD31 antibodies have not been reported. Here we describe for the first time array-CGH analysis of CNHC clusters cytomorphologically resembling CTMs. Array-CGH analysis revealed that the majority of the clusters of the CNHC-MF as well as the CNHC-UMF types did not show chromosomal aberrations and had a balanced genome. The DNA of one CNHC-UMF cluster isolated from a patient with pap. RCC and one CNHC-MF cluster from a patient with oncocytoma showed distinct chromosomal abnormalities on array-CGH which, however, did not match the aberrations found in the respective primary tumors (Figures 9 and 10, respectively) nor in a copy number aberration database (http://www.progenetix.net) [34]. It might be speculated that these CNHC clusters represent true CTMs. The reason for the differences of the chromosomal aberrations in the CNHCs and the respective tumors is not known, however, tumor heterogeneity [52] might be one of the factors responsible.

Data from animal models and patients with non-small-cell lung cancer indicate that cellular aggregates in blood may consist of CTCs associated with non-neoplastic cells like stromal cells which could enhance CTC survival and the metastatic process [23,53]. It has been estimated that array-CGH can detect gains and losses in mixed populations of tumor and non-neoplastic cells, if more than 20-25% of the population consist of tumor cells [54,55]. Therefore we cannot exclude that the CNHC of -MF or -UMF types without chromosomal aberrations described in our study also contain low numbers of CTCs that were not detectable by array-CGH.

Our results indicate that CNHC can be isolated from blood of patients with renal tumors using the ScreenCell® system. The majority of the isolated clusters may be of endothelial origin as indicated by positive staining with CD31 antibodies. These putative endothelial cell aggregates have hitherto not been reported in patients with renal tumors. It might be speculated that they mirror active angiogenesis in the tumors or during wound healing after surgery [56]. Increased numbers of CECs, probably shed from activated or damaged tumor vessel walls [57,58] and circulating endothelial progenitor cells derived from the bone marrow have been described in the blood of cancer patients and may importantly contribute to cancer growth and metastasis (reviewed in [58-60]). CECs are also found in several other clinical syndromes with vascular injury as well as in response to chemotherapy and anti-angiogenic treatment [61,62]. Therefore CECs and/or endothelial progenitor cells are considered biomarkers of vascular damage. Recently published data suggest CECs as prognostic [63] as well as predictive markers for response to anti-angiogenic therapy in prostate [64] and metastatic renal cell cancer [65-67]. However, their application as biomarkers in clinical practice has been limited by the difficulty to reliably detect them by flow cytometry [68]. Although limited by the relatively small number of cases analysed, the results of our study might indicate that detection of CECs may be facilitated by filtration based and immunocytochemical augmented methods. In this respect it is interesting that we detected a significant increase of the percentage of blood samples positive for all types of CNHCs one day after surgery as compared during surgery. This was also found for CNHC-UMF and –BF-positive samples, but not CNHC-MF-containing samples eight days after surgery.

## Conclusions

For patients with renal tumors cytomorphological classification alone seems not to be sufficient to allow for reliable distinction of epithelial CNHCs - the putative CTCs or CTMs - from endothelial CNHCs. As also suggested by others, reliable detection of CTCs or CTMs should thus be confirmed by immunocytochemical and/or molecular biological methods [17,69].

## Competing interests

The authors declare that they have no competing interests.

## Authors’ contributions

CL, PS, RZ, and BH designed the study. Data was collected and assembled by AE, TK, EZ, JH and CL. The interpretation and analysis was performed by AE, TK, CL, PS, BH, JBG, RR, JH. The samples and patient data were retrieved by KF, KKK, RZ, HP and RS. The manuscript was drafted by AE, CL, PS. All authors read and approved the final manuscript.

## Supplementary Material

Additional file 1: Movie 1 An exemplary laser capture microdissection. Click here for file

Additional file 2: Table S1 Correlation of tumor size and number of CNHCs. Click here for file

Additional file 3: Table S2 Relationship of tumor grade and number of CNHCs. Click here for file

Additional file 4: Table S3 Relationship of venous invasion and number of CNHCs. Click here for file

Additional file 5: Figure S1 Array-CGH profiles of the DNA of a clear cell RCC and the respective CNHCs or CNHCs derived from a patient with a cystic nephroma. The DNA of the clear cell RCC of patient #30 reveals gains of 5q, 7 and losses of 3p, changes commonly observed in clear cell RCC (red profile). The array-CGH profile of the DNA of two CNHC-UMF clusters (photograph A and B) of patient #30 represent with balanced genomes (profile A and B). The array-CGH profile of the two CNHC-UMF clusters (photograph C and D) revealed balanced genomes (profile C and D). All clusters were hematoxylin stained. The DNA of the respective renal tissue was not suited for array-CGH analysis due to insufficient quality. Gains and losses of the X- and Y-chromosomes do not reflect true copy number variations. They result from differences between the sex of the reference and the samples DNA (i.e. male patient DNA was hybridized against a female reference DNA thereby resulting in a loss of X and gain of Y chromosome and vice versa). Bars above the x-axis are considered to be gains, below the x-axis losses of DNA. Click here for file
