# Supplementary material for: Are morphological criteria sufficient for the identification of circulating tumor cells in renal cancer?
Source: J Transl Med. 2013 Sep 17;11:214. doi: 10.1186/1479-5876-11-214 (PMC3848446; doi:10.1186/1479-5876-11-214)
Supplement: Additional file 2: Table S1 — Correlation of tumor size and number of CNHCs. [file 1479-5876-11-214-S2.docx]

**Additional file 2: Table S1. Correlation of tumor size and number of CNHCs.**

| **Spearman-Rho** |  | **Time point A** | | |
| --- | --- | --- | --- | --- |
|  |  | Number of  CNHC-MF | Number of  CNHC-UMF | Number of  CNHC-BF |
| **Tumor size** | **Correlation coefficient** | -0.182 | -0.254 | 0.305 |
|  | **p-value (2-sided)** | 0.275 | 0.123 | 0.063 |
|  | **Number of Patients** | 38 | 38 | 38 |
|  |  | **Time point B** | | |
|  |  | Number of  CNHC-MF | Number of  CNHC-UMF | Number of  CNHC-BF |
| **Tumor size** | **Correlation coefficient** | 0.216 | 0.043 | 0.109 |
|  | **p-value (2-sided)** | 0.180 | 0.793 | 0.504 |
|  | **Number of Patients** | 40 | 40 | 40 |
|  |  | **Time point C** | | |
|  |  | Number of  CNHC-MF | Number of  CNHC-UMF | Number of  CNHC-BF |
| **Tumor size** | **Correlation coefficient** | 0.141 | 0.071 | -0.138 |
|  | **p-value (2-sided)** | 0.420 | 0.687 | 0.430 |
|  | **Number of Patients** | 35 | 35 | 35 |
|  |  | **Time point D** | | |
|  |  | Number of  CNHC-MF | Number of  CNHC-UMF | Number of  CNHC-BF |
| **Tumor size** | **Correlation coefficient** | -0.025 | 0.184 | -0.130 |
|  | **p-value (2-sided)** | 0.899 | 0.338 | 0.502 |
|  | **Number of Patients** | 29 | 29 | 29 |

There is no statistical significant correlation between tumor size and the number of detected CNHCs.
