# Supplementary material for: Are morphological criteria sufficient for the identification of circulating tumor cells in renal cancer?
Source: J Transl Med. 2013 Sep 17;11:214. doi: 10.1186/1479-5876-11-214 (PMC3848446; doi:10.1186/1479-5876-11-214)
Supplement: Additional file 3: Table S2 — Relationship of tumor grade and number of CNHCs. [file 1479-5876-11-214-S3.docx]

**Additional file 3: Table S2. Relationship of tumor grade and number of CNHCs.**

|  | **CNHC type** | **Fuhrman Grade** | **Number of**  **Patients** | **Number of CNHCs per 8ml (Median)** | **p-Value**  **(Chi-squared test)** |
| --- | --- | --- | --- | --- | --- |
| **Time point A** | CNHC-MF | 1 | 5 | 0 | 0.993 |
|  |  | 2 | 15 | 0 |  |
|  |  | 3 | 8 | 0 |  |
|  | CNHC-UMF | 1 | 5 | 0 | 0.312 |
|  |  | 2 | 15 | 0 |  |
|  |  | 3 | 8 | 3 |  |
|  | CNHC-BF | 1 | 5 | 0 | 0.486 |
|  |  | 2 | 15 | 0 |  |
|  |  | 3 | 8 | 0 |  |
| **Time point B** | CNHC-MF | 1 | 6 | 0 | 0.823 |
|  |  | 2 | 16 | 0 |  |
|  |  | 3 | 8 | 0 |  |
|  | CNHC-UMF | 1 | 6 | 0 | 0.623 |
|  |  | 2 | 16 | 0 |  |
|  |  | 3 | 8 | 0 |  |
|  | CNHC-BF | 1 | 6 | 0 | 0.877 |
|  |  | 2 | 16 | 0 |  |
|  |  | 3 | 8 | 0 |  |
| **Time point C** | CNHC-MF | 1 | 6 | 1 | 0.143 |
|  |  | 2 | 11 | 0 |  |
|  |  | 3 | 8 | 2 |  |
|  | CNHC-UMF | 1 | 6 | 0.5 | 0.997 |
|  |  | 2 | 11 | 0 |  |
|  |  | 3 | 8 | 0 |  |
|  | CNHC-BF | 1 | 6 | 5 | 0.536 |
|  |  | 2 | 11 | 0 |  |
|  |  | 3 | 8 | 4 |  |
| **Time point D** | CNHC-MF | 1 | 5 | 0 | 0.327 |
|  |  | 2 | 9 | 0 |  |
|  |  | 3 | 5 | 0 |  |
|  | CNHC-UMF | 1 | 5 | 12 | 0.130 |
|  |  | 2 | 9 | 0 |  |
|  |  | 3 | 5 | 6 |  |
|  | CNHC-BF | 1 | 5 | 12 | 0.053 |
|  |  | 2 | 9 | 0 |  |
|  |  | 3 | 5 | 0 |  |

There is no statistical significant correlation between the tumor grade and the number of detected CNHCs.
