# Supplementary material for: Are morphological criteria sufficient for the identification of circulating tumor cells in renal cancer?
Source: J Transl Med. 2013 Sep 17;11:214. doi: 10.1186/1479-5876-11-214 (PMC3848446; doi:10.1186/1479-5876-11-214)
Supplement: Additional file 4: Table S3 — Relationship of venous invasion and number of CNHCs. [file 1479-5876-11-214-S4.docx]

**Additional file 4: Table S3. Relationship of venous invasion and number of CNHCs.**

|  | **CNHC type** | **Venous**  **Invasion** | **Number of**  **Patients** | **Number of CNHCs per 8ml (Median)** | **p-Value**  **(Mann-Whitney-U test)** |
| --- | --- | --- | --- | --- | --- |
| **Time point A** | CNHC-MF | No | 30 | 0 | 0.836 |
|  |  | Yes | 6 | 0 |  |
|  | CNHC-UMF | No | 30 | 0 | 0.825 |
|  |  | Yes | 6 | 0 |  |
|  | CNHC-BF | No | 30 | 0 | 0.594 |
|  |  | Yes | 6 | 0 |  |
| **Time point B** | CNHC-MF | No | 32 | 0 | 1.000 |
|  |  | Yes | 6 | 0 |  |
|  | CNHC-UMF | No | 32 | 0 | 0.353 |
|  |  | Yes | 6 | 0 |  |
|  | CNHC-BF | No | 32 | 0 | 1.000 |
|  |  | Yes | 6 | 0 |  |
| **Time point C** | CNHC-MF | No | 28 | 0 | 0.018 |
|  |  | Yes | 5 | 11 |  |
|  | CNHC-UMF | No | 28 | 0.0 | 0.131 |
|  |  | Yes | 5 | 9.6 |  |
|  | CNHC-BF | No | 28 | 0 | 0.631 |
|  |  | Yes | 5 | 0 |  |
| **Time point D** | CNHC-MF | No | 25 | 0 | 1.000 |
|  |  | Yes | 2 | 1 |  |
|  | CNHC-UMF | No | 25 | 2 | 0.396 |
|  |  | Yes | 2 | 0 |  |
|  | CNHC-BF | No | 25 | 0 | 0.481 |
|  |  | Yes | 2 | 0 |  |

There is a statistical significant correlation between venous invasion and the number of detected CNHC-MF at time point C.
