# Supplementary figures and images for: Are morphological criteria sufficient for the identification of circulating tumor cells in renal cancer?
Source: J Transl Med. 2013 Sep 17;11:214. doi: 10.1186/1479-5876-11-214 (PMC3848446; doi:10.1186/1479-5876-11-214)

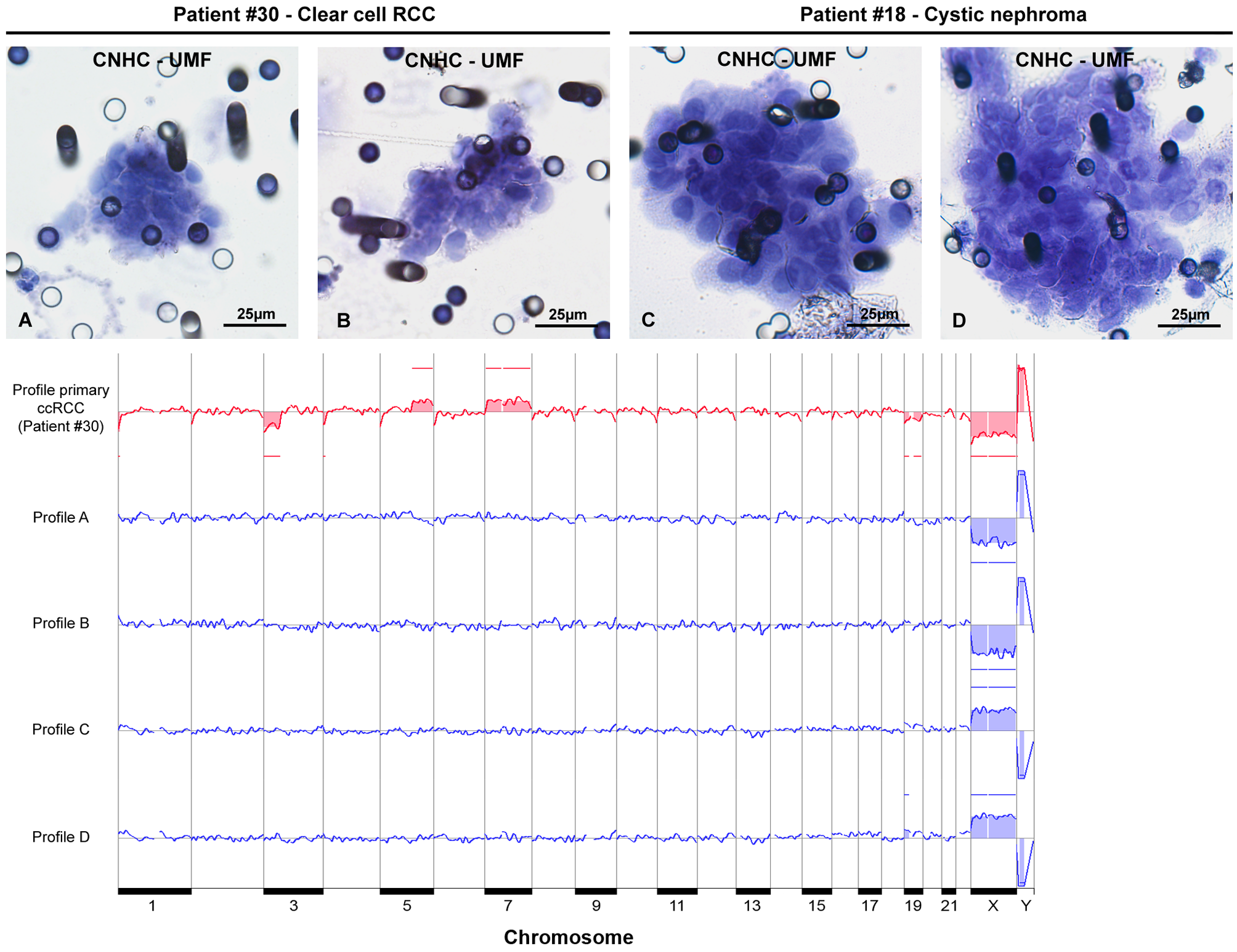

Supplement: Additional file 5: Figure S1 — Array-CGH profiles of the DNA of a clear cell RCC and the respective CNHCs or CNHCs derived from a patient with a cystic nephroma. The DNA of the clear cell RCC of patient #30 reveals gains of 5q, 7 and losses of 3p, changes commonly observed in clear cell RCC (red profile). The array-CGH profile of the DNA of two CNHC-UMF clusters (photograph A and B) of patient #30 represent with balanced genomes (profile A and B). The array-CGH profile of the two CNHC-UMF clusters (photograph C and D) revealed balanced genomes (profile C and D). All clusters were hematoxylin stained. The DNA of the respective renal tissue was not suited for array-CGH analysis due to insufficient quality. Gains and losses of the X- and Y-chromosomes do not reflect true copy number variations. They result from differences between the sex of the reference and the samples DNA (i.e. male patient DNA was hybridized against a female reference DNA thereby resulting in a loss of X and gain of Y chromosome and vice versa). Bars above the x-axis are considered to be gains, below the x-axis losses of DNA. [file 1479-5876-11-214-S5.tiff]
